# Faculty perspectives on the use of standardized versus non-standardized oral examinations to assess medical students

**DOI:** 10.5116/ijme.5b96.17ca

**Published:** 2018-09-29

**Authors:** Natasha Johnson, Holly Khachadoorian-Elia, Celeste Royce, Carey York-Best, Katharyn Atkins, Xiaodong P. Chen, Andrea Pelletier

**Affiliations:** 1 Department of Obstetrics, Gynecology and Reproductive Biology, Harvard Medical School, USA

**Keywords:** Standardized oral examination, obstetrics and gynecology, assessment

## Abstract

**Objectives:**

To determine if faculty perceive standardized oral examinations to be more objective and useful than the non-standardized format in assessing third-year medical students’ learning on the obstetrics and gynecology rotation.

**Methods:**

Obstetrics and gynecology faculty at three teaching hospitals were sampled to complete a survey retrospectively comparing the standardized oral examination (SOE) and non-standardized or traditional oral examinations (TOE).  A Likert scale (0-5) was used to assess satisfaction, objectivity, and usefulness of SOE and TOE.  Wilcoxon signed rank test was performed to compare median Likert scale scores for each survey item. A Spearman’s correlation coefficient was used to investigate the relationship between the perceived level of objectivity and SOE characteristics. For qualitative measures, content analysis was applied.

**Results:**

Sixty-six percent (n=25) of eligible faculty completed the survey. Faculty perceived the standardized oral examination as significantly more objective compared with the non-standardized (z=-3.15, p=0.002). Faculty also found SOE to be more useful in assessing overall clerkship performance (z=-2.0, p<0.05). All of the survey participants were willing to administer the standardized examination again. Faculty reported strengths of the SOE to be uniformity, fairness, and ease of use. Major weaknesses reported included inflexibility and decreased ability to assess students’ higher order reasoning skills.

**Conclusions:**

Faculty found standardized oral examinations to be more objective in assessing third-year medical students’ clinical competency when compared with a non-standardized approach. This finding can be meaningfully applied to medical education programs internationally.

## Introduction

The oral examination is commonly used to assess clinical knowledge and skills in both undergraduate and postgraduate medical education.  Given its apparent face validity, it is thought to be an effective way of assessing clinical competencies, including knowledge, communication skills, and critical thinking.  Although the oral examination has a long history in the professional development of physicians, concerns and fundamental questions remain about its use, the content validity, and the inter-rater reliability.^1^^-^^4^ In an oral examination, the trainee interacts with the examiner and is assessed based on answers provided to the questions asked.  Unconscious biases may influence the trainee's scores during the examination.  Common criticisms of oral examinations are the inherent variability and inconsistency associated with its subjective nature.^5^^-^^6^ Standardization of the oral examination content and grading rubric has been statistically shown to improve the objectivity of the oral examination.^7^^-^^9^

The effect of oral examinations on medical trainees (medical students and residents) and their academic performance has been investigated in many medical specialties, including surgery and internal medicine, but little has been reported in obstetrics and gynecology (OB/GYN) undergraduate medical education.^10^^-^^12^ Zahn and colleagues reported that OB/GYN rotations commonly require medical students to take an oral examination.^13^ In the OB/GYN rotations at Brigham and Women’s Hospital, Massachusetts General Hospital, and Beth Israel Deaconess Medical Center, all affiliated with Harvard Medical School, we have used a traditional non-standardized oral examination (TOE) as one summative assessment contributing to a final rotation grade for over twenty years. With TOE, the faculty examiner asks unscripted, non-standardized questions based on the content of the trainee’s submitted patient case list and faculty examiner’s knowledge of medical student educational objectives for the OB/GYN clerkship.  Although the TOE format has been shown to evaluate clinical knowledge and application among OB/GYN medical students, The Accreditation Council on Graduate Medical Education (ACGME) has suggested the standardized oral examination (SOE) as one of the assessment tools for graduate medical education.^14^^-^^15^ From OB/GYN students’ perspective, the SOE has been reported to be an effective alternative method to assess students’ clinical reasoning and contributes to students’ preparation for the written examination.^16^

Our study aimed to assess faculty perception of objectivity of our oral examination given in a standardized fashion versus the traditional non-standardized approach.  We replaced the TOE with a new SOE and implemented it in a pilot study in 2015. We investigated faculty’s satisfaction, acceptance of and perceived usefulness of using an SOE to assess third-year medical students’ learning and clinical competency during the OB/GYN rotation.

## Methods

### Educational contents and setting

An SOE was pilot-implemented within the OB/GYN rotations at Brigham and Women’s Hospital, Massachusetts General Hospital, and Beth Israel Deaconess Medical Center in 2015. These three institutions are teaching hospitals affiliated with Harvard Medical School. OB/GYN rotations are six weeks in length for third-year Harvard Medical School students. The goals of an SOE are to assess the ability of each student to understand and discuss the pathophysiology, differential diagnosis, diagnostic evaluation and treatment of patient cases, as well as to demonstrate the student’s presentation and clinical reasoning skills. Students are asked to prepare four cases encountered during their clerkship and complete a structured case list. Students select one case from each of three categories (benign gynecology, gynecologic subspecialties, and obstetrics) a fourth ambulatory case from any of the three categories. The categories were determined by the OB/GYN medical school leadership and correspond to the Association of Professors of Gynecology and Obstetrics (APGO) 10th Edition Medical Student Objectives. Each student has two 20-minute oral exams with trained faculty examiners.

Each faculty member asks the student to briefly present the patient (2-3 minutes) followed by a 7-8 minute question and answer period.  Two cases are covered in each oral exam respectively.  We also developed a new SOE toolbox for faculty examiners based on our previous study which included  the  creation of   standardized  questions  based on APGO teaching cases. The standardized questions consist of basic questions that students are expected to answer satisfactorily in order to pass the oral examination and also more advanced questions that examiners can select depending on students’ performance on basic questions.^17^ Each examiner fills out an evaluation form at the conclusion of the exam. A faculty development resource, which consists of a slide presentation introducing the SOE format, directions for asking standardized questions based on topic selection (including information to modify standard questions to reflect the specifics of the case), the SOE grading instrument, how to differentiate student performance utilizing sample questions/answers, and videos of oral examinations using the new SOE format, was provided to all faculty members.

### Study design and data collection

Our study was a survey-based quantitative and qualitative study. In 2016, thirty-eight OB/GYN faculty who administered at least one SOE during 2015-2016, were sampled and invited to complete an SOE survey one year after its pilot-implementation. We developed the survey questionnaires based on current best practices in survey design and our study objective.^17^^-^^18^ Expert validation was applied to ensure the face and content validity: three content experts who had concrete knowledge and experience in OB/GYN clerkship teaching assessed the survey items’ clarity and relevance to ensure the construct. We then conducted cognitive pretesting with one faculty to ensure participants would interpret the survey items in the manner that we intended. The research team discussed until reaching final consensus and then finalized the survey questionnaire. The final survey instrument included six items measuring the faculty members’ satisfaction and perceived usefulness of SOE using a 5-point-Likert scale, as well as two free-text questions inquiring about the strengths and weaknesses of SOE (Appendix 1). Faculty were asked to rate the perceived objectivity, satisfaction (required time commitment, and willingness of participation), and usefulness in assessing students’ competencies and clerkship performance using SOE, as well as provide retrospective ratings for the TOE.  Two open-ended questions were also asked to generate additional free-text commentary on the strengths and weaknesses of the oral examinations. Faculty participation was voluntary. Anonymous survey responses were collected through the online survey tool. The study was approved by the institutional review board (IRB) at all three participating institutions.

### Statistical analysis

All analyses were conducted using STATA version 15.1. For the 5-point-Likert scale survey items, we reported proportions. We used the Wilcoxon signed rank test to look at the differences in median Likert Scale score between the SOE and the TOE for each survey question. Median values, z scores, and p values were reported for the Wilcoxon test. Multivariate analysis was used to examine the relationship between the level of objectivity and the other six variables of the SOE (assessment of communication skills, clinical knowledge, knowledge application, clinical reasoning, professionalism, and overall clerkship performance). Based on these results, we reported correlation coefficients and p values using Spearman’s correlation coefficient. The non-parametric Spearman’s correlation and the Wilcoxon signed rank test was used because most data did not have a normal distribution and because the Likert scale data were considered ordinal. P-values less than 0.05 was considered statistically significant. Content analysis was utilized to examine the responses to open-ended questions about the weaknesses and strengths of SOE. Two authors coded the comments separately to identify any patterns or evidence of change over time in faculty members’ descriptions. The authors then discussed and reached consensus on the themes.

## Results

Among the 38 faculty members who were eligible for participation, 66% (n=25) completed the SOE survey. The majority of faculty participants (80%) reported administering SOE 1-5 times (as compared with >5 times) during 2015-2016. Twenty of the 25 participants (80%) had administered the TOE before launching the SOE.

Table 1 shows that, overall, 88% of faculty reported being “satisfied or very satisfied” with the SOE as compared to 85% with the TOE and 100% of faculty participants (n=25) indicated they would like to administer an SOE again. The level of objectivity reported as “objective or very objective” was 92% for SOE versus 60% for the TOE. When asked if SOE was a more objective way to assess students’ clinical knowledge and skills, 88% said yes. Faculty rated the level of usefulness higher for SOE on all items except communication and professionalism, where TOE slightly outperformed SOE.

As shown in Table 2, the median scores for objectivity were significantly higher for SOE with a median score of 5 versus TOE with a median score of 4 (z=-3.15, p=0.002). The SOE also scored significantly higher than a TOE in assessing overall clerkship performance (z=-2.00, p=0.046).

We conducted secondary data analysis to investigate further whether a higher level of objectivity perceived by faculty would associate with a higher perceived level of SOE’s usefulness. As shown in Table 3, faculty participants’ perceived level of objectivity of the SOE strongly correlated with the SOE’s perceived usefulness in assessing students’ clinical knowledge (correlation coefficient 0.75, p=0.03) and knowledge application (correlation coefficient=0.77, p< 0.001). However, the faculty participants’ perceived level of objectivity of the SOE only demonstrated a weak-to-moderate association with the usefulness of the other four SOE items (assessment of communication skills, clinical reasoning, professionalism, and overall clerkship performance).

The majority of faculty participants completed the two free-text questions about the weaknesses and strengths of SOE (96%, n=24). Responses to the open-ended question “What do you think the strengths of the current oral examination are?” described specific strengths of an SOE, as well as a TOE (Table 4). Specific strengths of the SOE described by many faculty members included uniformity, fairness, and ease of use. Faculty participants reported the SOE’s major weaknesses as inflexibility and decreased ability to assess students’ higher-order reasoning skills by having to focus on standardized questions. Faculty participants noted strengths of the TOE including the ability to assess students’ reasoning skills, as well as to demonstrate students’ learning.  When asked about the weaknesses of the current oral exam, faculty participants described the TOE as lacking standards on grading, having strong individual variations, and time pressure.

## Discussion

Our findings suggest that, compared to TOE, the OB/GYN faculty perceived SOE was a more objective assessment tool to evaluate medical students’ learning on the clerkship without the additional required time commitment. OB/GYN faculty examiners perceive the SOE as more objective and useful in assessing clerkship students’ clinical knowledge, knowledge application, clinical reasoning, and overall clerkship performance than the TOE. Results of perceived improvement in SOE’s level of objectivity is consistent with those reported by Crisostomo and others that standardization of the oral exam content and grading rubric could improve the subjective nature of oral examinations.^7^^-^^9^

Standardization and comparability are critically important in medical schools which have multi-site clerkships, such as ours, to ensure compliance with The Liaison Committee on Medical Education (LCME) Standard 8.7, Comparability of Education/Assessment.^19^ The introduction of the Association of American Medical Colleges (AAMC) Entrustable Professional Activities (EPAs) is another effort to standardize student assessments.^20^ The SOE not only satisfies one of The LCME Assessment Standards (Standard 9.6, Setting Standards of Achievement), but also can be utilized as a tool to assess EPAs (specifically EPA 2, prioritize a differential diagnosis following a clinical encounter, EPA 3, recommend and interpret common diagnostic and screening tests, EPA 6, provide an oral presentation of a clinical encounter and EPA 7, form clinical questions and retrieve evidence to advance patient care).

Results from our study illustrate that faculty members’ perceived level of objectivity of the SOE strongly correlates with their perceived SOE’s abilities of assessing students’ clinical knowledge and knowledge application. However, the correlations between the level of objectivity and the SOE’s ability to assess communication skills and clinical reasoning were not statistically significant.

**Table 1:** Faculty perspective of characteristics of the traditional oral examination (TOE) and the standardized oral examination (SOE)

Survey Item	TOE (n=20) % (n)	SOE (n=25) % (n)
Number of times administered an oral exam		
1-5	35 (7)	80 (20)
6-10	30 (6)	8 (2)
11-15	5 (1)	8 (2)
> 15	30 (6)	4 (1)
Satisfaction about the required time commitment		
Satisfied and Very Satisfied	85 (17)	88 (21)
Neutral	15 (3)	8 (2)
Dissatisfied and Very dissatisfied	0 (0)	4 (1)
Level of objectivity of oral exam in assessing students’ clinical knowledge and skills		
Objective and Very Objective	60 (12)	92 (23)
Neutral	25 (5)	0 (0)
Subjective and Very subjective	15 (3)	8 (2)
Level of usefulness of oral exam in assessing:		
Communication		
Extremely useful	50 (10)	48 (12)
Moderately useful and Somewhat useful	45 (9)	40 (10)
Slightly useful and Not at all useful	5 (1)	12 (3)
Clinical knowledge		
Extremely useful	25 (5)	44 (11)
Moderately useful and Somewhat useful	65 (13)	48 (12)
Slightly useful and Not at all useful	10 (2)	8 (2)
Knowledge application		
Extremely useful	25 (5)	48 (22)
Moderately useful and Somewhat useful	70 (14)	48 (22)
Slightly useful and Not at all useful	5 (1)	4 (1)
Clinical reasoning		
Extremely useful	35 (7)	40 (10)
Moderately useful and Somewhat useful	55 (11)	56 (14)
Slightly useful and Not at all useful	10 (2)	4 (1)
Professionalism		
Extremely useful	30 (6)	24 (6)
Moderately useful and Somewhat useful	35 (7)	48 (12)
Slightly useful and Not at all useful	35 (7)	28 (7)
Student overall clerkship performance^*^		
Extremely useful	11 (2)	22 (5)
Moderately useful and Somewhat useful	72 (13)	69 (16)
Slightly useful and Not at all useful	17 (3)	9 (2)
Oral exam with standardized questions would be a more objective way to assess students’ clinical knowledge and skills?		
Yes	N/A	88 (22)
Would like to administer the oral exam again?		
Yes	N/A	100 (25)

*There were missing data for TOE and SOE for the item asking about overall clerkship performance.

**Table 2:** Faculty perspective of the standardized oral examination (SOE) compared to the traditional oral examination (TOE)

Faculty perspective	SOE^*^ Median (25-75% IQR)	TOE Median (25-75% IQR)	Z Score^**^	p value
Satisfaction w/Required Time Commitment	5 (4-5)	4 (4-5)	-1.273	0.20
Level of Objectivity	5 (4-5)	4 (3-4)	-3.145	0.002
Assess Communication	4 (4-5)	4.5 (4-5)	0.607	0.55
Assess Clinical Knowledge	4 (4-5)	4 (3-4.5)	-1.944	0.05
Assess Knowledge Application	4 (4-5)	4 (3-4.5)	-1.778	0.08
Assess Clinical Reasoning	4 (4-5)	4 (4-5)	-0.444	0.66
Assess Professionalism	3 (2-4)	3 (2-5)	0.607	0.54
Assess Overall Clerkship Performance	4 (3-4)	4 (3-4)	-2.000	0.046

^*^n=20 for paired data analysis. Ratings were given as median; 5 = very satisfied/ extremely useful, 1 = very dissatisfied/not at all useful.

^**^standard normal distributed z value to test for significance of median Likert scale scores between TOE and SOE.

One possible reason is that standardization of questions limits faculty examiners’ flexibility to adjust questions based on the case scenario. As some faculty participants commented on the weaknesses of an SOE, “It's not always possible to stay with the ‘standard’ questions, as the clinical case does not always lend itself to that.” Another possible reason is that standardization of questions restricts faculty examiners’ style of asking questions. In our study, faculty participants perceived the TOE was slightly more useful in assessing students’ communication skills than the SOE; some of them described the flexibility of tailoring the oral exam questions based on how students respond throughout the case presentation as valuable. 

**Table 3:** Correlation between the level of perceived objectivity and the characteristics of the standardized oral examination reported in the post-implementation SOE survey by the faculty (N=20)

Characteristics of standardized oral examination	Correlation Coefficient^*^	p value
Assess Knowledge Application	0.77	<0.001
Assess Clinical Knowledge	0.75	0.03
Assess Professionalism	0.48	0.01
Assess Clerkship Overall Performance	0.40	0.03
Assess Communication	0.34	0.22
Assess Clinical Reasoning	0.14	0.27

*Spearman correlation; strong correlation >0.70.

**Table 4:** Themes of the strengths and weaknesses of the traditional oral examination and the standardized oral examination

Themes	Traditional oral examination^*^	Standardized oral examination^¶^
Strengths Examples of faculty comments	The ability of assessing students’ reasoning skills “Oral exams allow you to assess their (students’) reasoning and communication skills, and to converse with them.” *“I believe the oral exams prepare students for clinical reasoning needed to practice medicine.”* Demonstrates students’ learning “Gives students an opportunity to show their skills in a different and more "applicable" way.” “They (students) present their own cases and are usually knowledgeable about the case.”	Uniformity “Consistency in general clinical knowledge assessment.” “Uniform question list for representative cases that allows more direct comparison of examinees’ knowledge base” Fairness “Keeps the examiner grounded as to the level of knowledge and reasoning that should be expected for a medical student. Fairness.” “Improves examiner organization and consistency. Standardization attempts to eliminate unconscious bias and allows there to be a system of fair and equitable evaluation.” Easy to use “Helps less experienced faculty examiners administer the oral exam more easily.”
Weaknesses Examples of faculty comments	No grading standards “Somewhat subjective” “Grading sometimes seems to vary among examiners” “Lack of standardization” Strong individual variations “Variation in examiner skills, biases and techniques.” “The variability in each student’s case list and presentation.” Time pressure “I think it takes a lot of time, which is something most of us have little of.” “Takes students away from the wards an additional 2-3 hours per block”	Inflexible “The standardized questions aren't always appropriate for the case the student has listed.” “At times the students’ choice of patient does not quite fit into the category of questions so I end up not completely sticking to the standardized questions.” “The flow of the oral exam as a "conversation" is made more challenging by the standardized questions -- this is a minor weakness.” The ability of assessing students’ higher order reasoning skills by having to focus on standardized questions “Focusing on standardized questions left less opportunity to assess (students’) higher-order clinical reasoning.”

*Faculty comments from pre-implementation survey; ¶Faculty comments from post-implementation survey.

This exemplifies the need to reinforce a certain level of flexibility into the SOE (e.g., instructions for faculty to modify standardized questions to reflect specifics of the cases) and guidelines to empower faculty examiners to assess students’ clinical reasoning skills further when appropriate. Future study is needed to explore an optimal solution to fulfill this need. In addition, few faculty reported the TOE was extremely useful in assessing student’s overall clerkship performance. This reflects the grading rubric at our institutions, where students’ clinical performance over the 6-week rotation accounts for 70% of the clerkship grade in our institutions and this is the major determinant of overall performance.

Our study has several limitations. First, our sample size of the SOE survey is small. In addition, participants are from 3 teaching hospitals affiliated with a single medical school in the same geographic region; results may therefore not be generalizable to all OB/GYN clerkship. Second, the survey response rate was somewhat low (<70%), and the non-response bias might impact current results. Third, these results represent faculty examiners’ self-reported opinions and do not measure clerkship students’ actual competencies or academic outcomes. Future research would benefit from the incorporation of clerkship students’ perspective and performance into faculty members’ perspective of the SOE to further refine its design and implementation. Students’ performance on the SOE relative to other clerkship performance metrics would also be interesting to study.

## Conclusions

OB/GYN faculty examiners perceive the SOE as more objective and outperforming the TOE in assessing medical students’ clinical knowledge, knowledge application, clinical reasoning, and overall clerkship performance. Programs in medical education are encouraged to introduce the standardized oral examination to their faculty and/or replace the traditional non-standardized oral examinations with the SOE to increase objectivity in assessing medical students’ learning and performance.  Future studies should include evaluation of this assessment tool in surgery and other medical fields that routinely administer oral examinations in undergraduate and graduate education.  This finding can be applied internationally in the assessment of medical students’ clinical competency and critical thinking skills.

### Conflict of Interest

The authors declare that they have no conflict of interest.

## Supplementary material

Supplementary file 1 Appendix 1. OBGYN Oral Exam Online Survey
