# Supplementary material for: Faculty perspectives on the use of standardized versus non-standardized oral examinations to assess medical students
Source: Int J Med Educ. 2018 Sep 29;9:255–61. doi: 10.5116/ijme.5b96.17ca (PMC6387762; doi:10.5116/ijme.5b96.17ca)
Supplement: Supplementary file 1 — Appendix 1. OBGYN Oral Exam Online Survey [file ijme-9-255-S1.pdf]

Appendix 1

OBGYN Oral Exam Online Survey

1. Approximately how many times have you administered the oral exam to OB/GYN clerkship students?

Non-standardized	A) 1-5	B) 6-10	C) 11-15	D) >15	E) N/A
Standardized	A) 1-5	B) 6-10	C) 11-15	D) >15	

2. Please indicate your level of satisfaction about the required time commitment (both preparation and time to administer) for the oral exam:

Non-standardized	1. Very Dissatisfied	2. Dissatisfied	3. Neutral	4. Satisfied	5. Very Satisfied 6 N/A
Standardized	1. Very Dissatisfied	2. Dissatisfied	3. Neutral	4. Satisfied	5. Very Satisfied

3. Please indicate how objective you feel the oral exam is in assessing students' clinical knowledge and skills:

Non-standardized	1. Very subjective	2. Subjective	3. Neutral	4. Objective	5. Very objective	6. N/A
Standardized	1. Very subjective	2. Subjective	3. Neutral	4. Objective	5. Very objective	

4. Please rate the usefulness of the oral exam in assessing the students' performance and skills in the following areas:

Performance and Skills	Type of Oral Exam	1-Not at all useful<br>2-Slightly useful<br>3-Somewhat useful<br>4-Moderately useful<br>5-Extremely useful<br>N/A
Communication	Non-standardized	
	Standardized	
Level of clinical knowledge	Non-standardized	
	Standardized	
Level of clinical knowledge application	Non-standardized	
	Standardized	
Clinical reasoning	Non-standardized	
	Standardized	
Professionalism	Non-standardized	
	Standardized	
Student's overall performance in the OB/GYN clerkship	Non-standardized	
	Standardized	

5. Would you be willing to continue to administer the oral exam using the standardized exam to OB/GYN clerkship students again?
   Yes [ ] No [ ]

6. Do you think the standardized oral exam is a more objective way than the non-standardized exam to assess students' clinical knowledge and skills?
   Yes [ ] No [ ] Uncertain [ ] N/A did not administer non-standardized exam [ ]
   (if No, please explain)

7. What do you think the weaknesses/challenges of the standardized oral exam are?
   [Open comment]

8. What do you think the strengths of the standardized oral exam are?
   [Open comment]
